# CO_2_ removal to reach net zero warming of global methane and nitrous oxide emissions of livestock: Comparison of two metrics under different 2050 FAO scenarios

**DOI:** 10.1371/journal.pone.0330379

**Published:** 2025-08-18

**Authors:** Fabio Correddu, Mondina Francesca Lunesu, Sara Sechi, Maria Francesca Caratzu, Giuseppe Pulina

**Affiliations:** Dipartimento di Agraria, University of Sassari, Sassari, Italy; Texas A&M University, UNITED STATES OF AMERICA

## Abstract

Achieving global climate targets requires accurate quantification of greenhouse gas (GHG) emissions and their implied impact on temperature. However, the choice of emission metric—particularly *between* Global Warming Potential over 100 years (GWP_100_) and Global Warming Potential Star (GWP*)—can significantly influence how emissions and their contributions to global warming are represented in climate assessments. While metrics do not alter physical temperature outcomes, they affect how emissions’ impacts are interpreted, which in turn influences carbon dioxide removal (CDR) estimates and mitigation strategies. Using FAO projections for global livestock emissions to 2050, we analyze how different metric choices affect estimates of the CDR required to offset methane (CH₄) emissions and achieve no additional warming condition. Our findings highlight that GWP_100_ can overestimate or underestimate the cumulative warming impact of CH₄ emissions under different emission trajectories, whereas GWP* provides a dynamic approach that better aligns with temperature goals*.* These differences have critical implications for climate policy, as they influence the perceived effectiveness of mitigation strategies and the allocation of CDR requirements. This study underscores the necessity of selecting appropriate metrics when designing climate mitigation frameworks, particularly for methane-intensive sectors like livestock, to ensure an accurate representation of their contribution to global temperature targets.

## Introduction

To achieve the goal of limiting global warming to +1.5°C above pre-industrial levels [1], countries and different productive sectors are called upon to make a big effort to reduce their Greenhouse Gases (GHG) emissions and improve removal by sinks. However, the GHG mitigation goal of achieving ‘a balance between anthropogenic emissions by sources and removals by sinks of greenhouse gases’ could be subject to interpretation. In particular, the implications for individual gases depend on the metrics used to relate them [2]. GHGs can be classified into two categories: long-lived climate pollutants (LLCPs), such as carbon dioxide (CO₂) and nitrous oxide (N₂O), and short-lived climate pollutants (SLCPs), such as methane (CH₄). The LLCPs accumulate in the atmosphere over centuries, and, to stop their warming effect, emissions must be reduced to net zero; the SLCPs have a shorter atmospheric lifetime because they are continuously removed. This means that a constant level of methane emissions can be compatible with a stabilization of global temperatures [3,4]. Thus, the relationship between cumulative greenhouse gas emissions and temperature outcomes differs between long-lived and short-lived climate pollutants. Cumulative CO₂ emissions exhibit a nearly linear relationship with temperature increase [5], whereas CH₄ does not follow this pattern due to its shorter atmospheric lifetime and continuous removal from the atmosphere. As a result, the use of GWP_100_ can overestimate the impact of CH₄ in decreasing emission scenarios and underestimate it in increasing emission scenarios [6].

A more accurate approach is the Global Warming Potential Star (GWP*), which better represents the impact of SLCPs like methane on temperature. This new metric has been recognized by the Intergovernmental Panel on Climate Change (IPCC) [5] as a more appropriate tool for understanding how different GHGs contribute to global warming over time. The Food and Agriculture Organization (**FAO**) [7] also recommends using GWP* to assess the warming effect of GHGs under historical and future emissions scenarios, particularly in livestock emissions research. The interpretation of net-zero and its implications for temperature goals depends on the metric used to equate the impact of different GHGs. As highlighted by Tanaka and O’Neill [8], the Paris Agreement’s net-zero emissions goal does not always align with temperature targets of 1.5°C or 2°C, particularly when different GHGs are aggregated using conventional metrics like GWP_100_, which may overestimate the contribution of short-lived pollutants to cumulative warming. Similarly, Fuglestvedt et al. [2] emphasize that different interpretations of net-zero CO₂-equivalent emissions lead to varying temperature outcomes, as conventional methods like GWP_100_ often assume linear accumulation rather than considering the distinct atmospheric behaviour of short-lived pollutants.

These different interpretations can also affect the estimation of residual emissions to be offset, which is crucial in context of temperature stabilization pathways. Residual emissions can be offset by removing CO_2_ from the atmosphere (carbon dioxide removal, **CDR**) through a set of techniques including afforestation and reforestation, land restoration and soil carbon sequestration, and direct air carbon capture and storage [5]. As reported by Brazzola et al. [9], CDR requirements are strongly dependent on the choice of metric, as applying GWP_100_ to methane-heavy sectors can miscalculate the actual CO₂ removal needed for temperature stabilization. These findings highlight the necessity of metric-aware climate strategies, particularly when designing offsetting policies and estimating CDR to achieve net-zero goals in methane-emitting sectors such as livestock. Indeed, applying GWP_100_ to methane-heavy sectors like livestock can misrepresent the actual amount of CO₂ removal required to meet climate targets. Brazzola et al. [9] reported that GWP* is more suitable for offsetting calculations, although it slightly overestimates the required CDR under sustained methane emissions scenarios.

Livestock is among the highest-emitting sectors in terms of GHG emissions, largely due to methane from enteric fermentation and manure management. Several studies indicate that the rising global demand for animal-source foods (ASF) will likely increase ruminant-derived emissions, posing challenges for net-zero climate goals [10–12]. However, most of these assessments rely on GWP_100_, which does not account for the different behaviour of methane compared to CO₂ and N₂O.

The FAO [13] recently evaluated potential pathways for reducing livestock-related emissions by 2050. Their report explores two scenarios based on the projected increase in animal protein demand by 2050: a business-as-usual (BAU) assessment, assuming continued emissions growth without mitigation strategies and, an alternative scenario, in which mitigation strategies (e.g., improved feed efficiency, manure management, and carbon sequestration) are implemented. Nonetheless, the calculation of the CDR required to achieve no additional warming depends critically on the choice of metric used to evaluate methane emissions.

This study applies both GWP_100_ and GWP* to FAO’s 2050 scenarios to determine how the choice of metric affects estimated temperature projections, and the estimated CDR required to achieve the temperature goal (no additional warming).

## Materials and methods

### Emissions and warming impact

This study uses FAOSTAT [14] as a source of historical GHG (CH_4_ and N_2_O) emissions data (1961–2021) from global livestock and the FAO report [13] for the projected emissions from global livestock systems by 2050.

**Fig 1:**
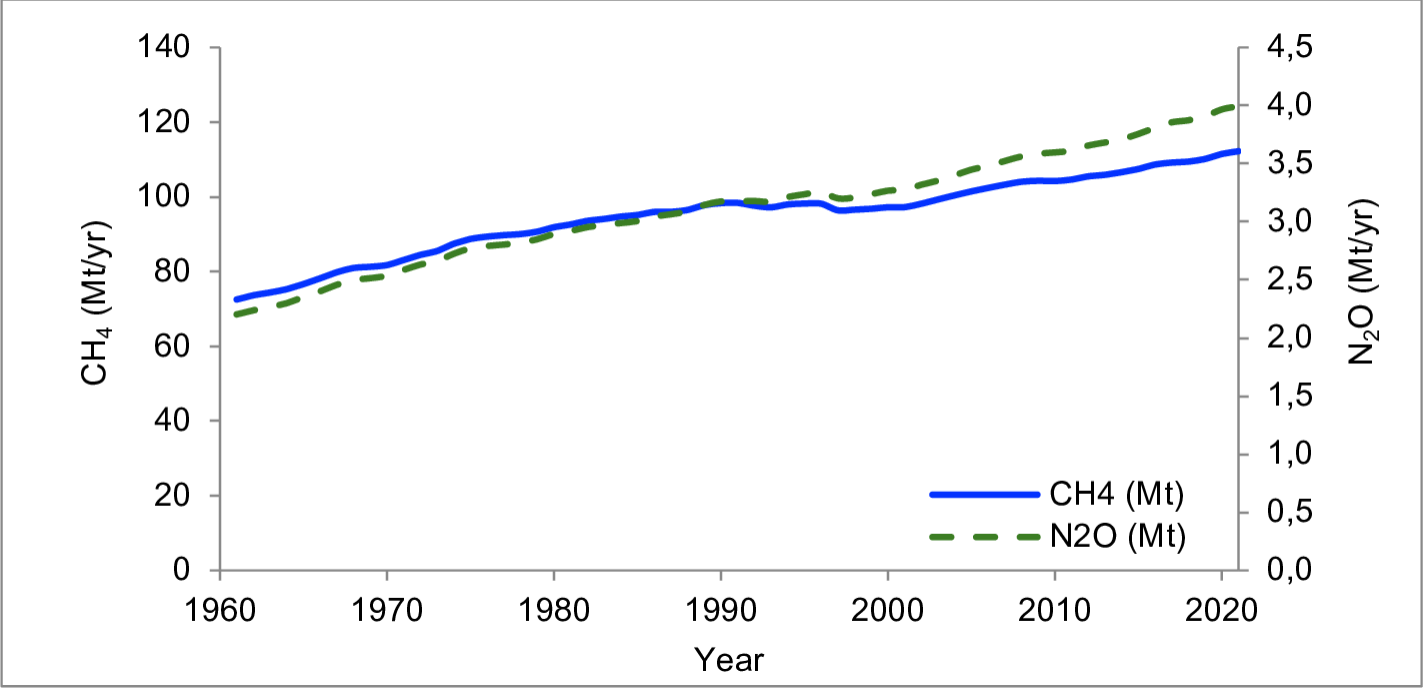
Annual CH_4_ (methane) and N_2_O (nitrous oxide) emissions (Mt/yr) from global livestock sector for the 1961-2021 period (FAOSTAT, 2024) [14].

Historical GHG emissions data are computed according to Tier 1 of the Intergovernmental Panel on Climate Change (IPCC) Guidelines for National Greenhouse Gas Inventories [15–19], with a global coverage for the period 1961–2021. Annual CH_4_ emissions include those deriving from enteric fermentation and manure management. To estimate total N_2_O emissions, annual emissions related to manure management, manure left on pasture, and manure applied to the soil provided by FAOSTAT [14] were considered. The Fig 1 reports the historical CH_4_ and N_2_O annual emission rates (1961–2020) for the global livestock sector.

**Fig 2:**
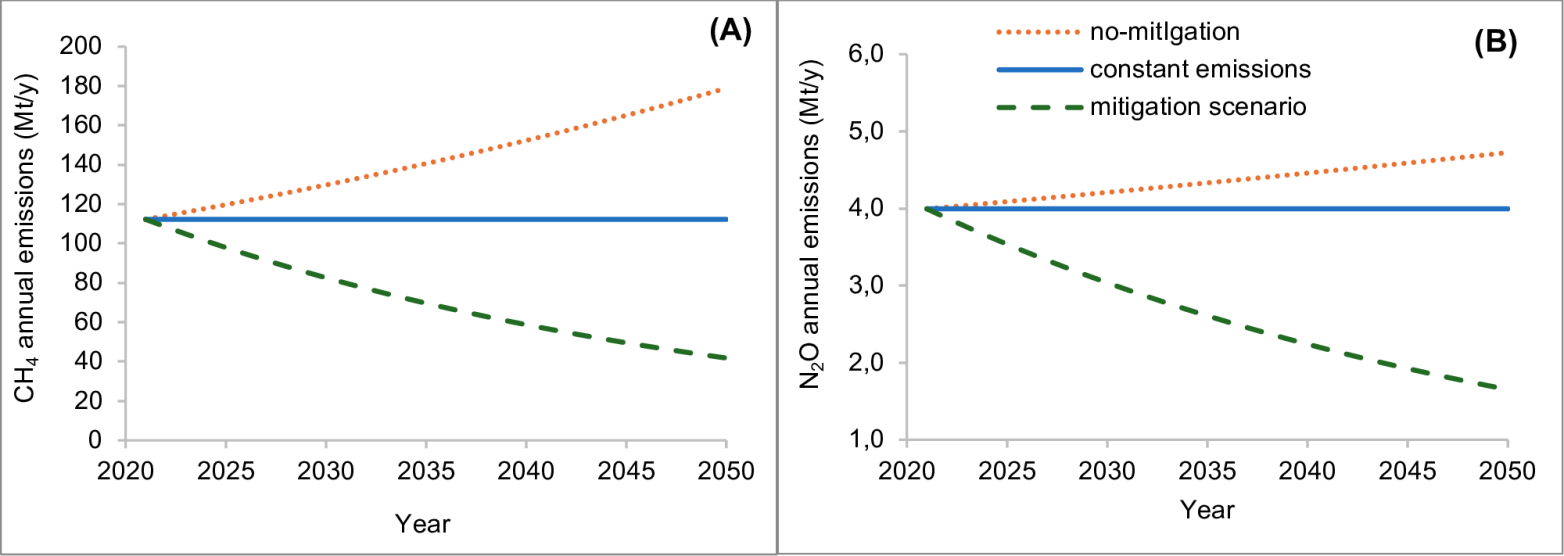
Projected annual CH_4_ (A) and N_2_O (B) emissions (Mt/yr) by 2050, based on FAO no-mitigation (increasing) and mitigation (decreasing) scenarios, and on a constant emission scenario.

Projected emissions (CH_4_ and N_2_O) from global livestock systems by 2050 reported by the FAO (2023) were used to assess two pathways: increasing and decreasing scenarios. The FAO [13] provides only the final estimated emissions for each scenario in 2050 and does not include annual emission trends. To include these scenarios into our analysis, we constructed annual emission trajectories assuming constant annual rates of variation to reach the final values of GHG emissions estimated for 2050 (Fig 2A for CH_4_ and Fig 2B for N_2_O).

i) The increasing scenario relates to a no-mitigation scenario, based on the FAO’s “projected 2050 emissions, no mitigation” [13]. This scenario accounts for additional emissions estimated for 2050, considering the global increase in demand for animal protein (21% by 2050) and assuming that no interventions will be adopted to reduce the GHG emissions. The trajectory was built considering the initial values of CH_4_ and N_2_O emissions (2021) and a constant rate of increase to reach the final values of estimated GHG emissions (2050).
ii) The decreasing scenario relates to the FAO’s “Low-emission pathways to 2050” [13], which is based on various interventions aimed at mitigating the livestock impact, while meeting the needs of the growing population, estimated with an increase of 21% of animal protein production by 2050. These mitigation interventions are the result of a comprehensive literature review reported in the FAO report [13] and include nutritional, health, and breeding interventions, as well as reducing food loss and waste, improvement of circular bioeconomies and manure management. The trajectory was built considering the initial values of CH_4_ and N_2_O emissions (2021) and a constant decreasing rate to reach the final values of GHG emissions estimated for 2050.
iii) In addition, we explore a constant pathway of emissions (constant scenario). This scenario is built on the hypothesis that CH_4_ and N_2_O emissions remain stable at their 2021 levels, and assuming no interventions adopted to reduce the GHG emissions.

Values of CH_4_ and N_2_O arising from the three scenarios were converted into CO_2_*e* following the standard IPCC GWP_100_ and, only for CH_4_, into CO_2_*we* according to the GWP* metric. The obtained values for CH_4_ and N_2_O were then aggregated into the two metrics to follow the trajectories, for annual and cumulative emissions.

To assess the implications of these emission pathways for temperature goal (no additional warming), we calculated the necessary carbon dioxide removal (CDR) required to offset non-CO₂ emissions under two different greenhouse gas equivalency metrics, GWP_100_ and GWP*.

#### Calculation of CO_2_*e.*

The CO_2_e of annual CH_4_ and N_2_O emissions for global livestock sector were calculated following the equation [1] of the IPCC [20], where E is the annual CH_4_ or N_2_O emission and GWP_H_ is the global warning potential of one ponderal unit CH_4_ or N_2_O in a time horizon (H) of 100 years, corresponding, respectively, to 28 and 265 units of CO_2_*e* [19]; we prefer to keep a slightly older version for comparability with previous GWP literature.

[1] GWP (CO_2_*e*) = E × GWP_H_

[20]

#### Calculation of CO_2_*we.*

The CO_2_*we* of annual CH_4_ emissions for global livestock sector were calculated following the equation [2] of Smith et al. [21], where ESLCP(t) is the annual CH_4_ emission rate for a considered year, and ESLCP(t-20) is the annual CH_4_ emission rate relative to 20 years before t.

[2] GWP* (CO_2_*we*) = GWP_100_ × [4.53 × E_SLCP_(t) – 4.25 × E_SLCP_(t-20)]

[21]

For N_2_O, the value of CO_2_*we* was set equal to that of CO_2_*e*. See Mc Auliffe et al. [22] for previous similar application of this equivalence, and Allen et al. [23] for a more mathematically precise approach and explanation.

#### Temperature outcomes.

The impact of GHG emissions on global temperature was assessed by calculating the warming effect, using the Transient Climate Response in Cumulative Emission (TCRE) coefficient. According to the IPCC report [5] each Tt of cumulative CO_2_ emission is likely to cause a 0.45°C (0.27–0.63°C) increase in global temperature. Then the approximate estimation of the warming effect (temperature change) can be obtained by multiplying the cumulative CO_2_ emissions (and by extension the cumulative CO_2_*e* or CO_2_*we*) by the TCRE [24].

#### Carbon dioxide removal.

We estimated the Carbon Dioxide Removal (CDR) required to offset global livestock emissions arising from the three scenarios, with the aim of reaching the goal of “net-zero warming” by 2050. It should be noted that there is no officially established “net zero warming” goal; rather, this concept was hypothesized in this work, with the aim of better exploring the different implications of using the two metrics when calculating the amount of emissions that need to be offset in order to achieve the climate goal.

**Fig 3:**
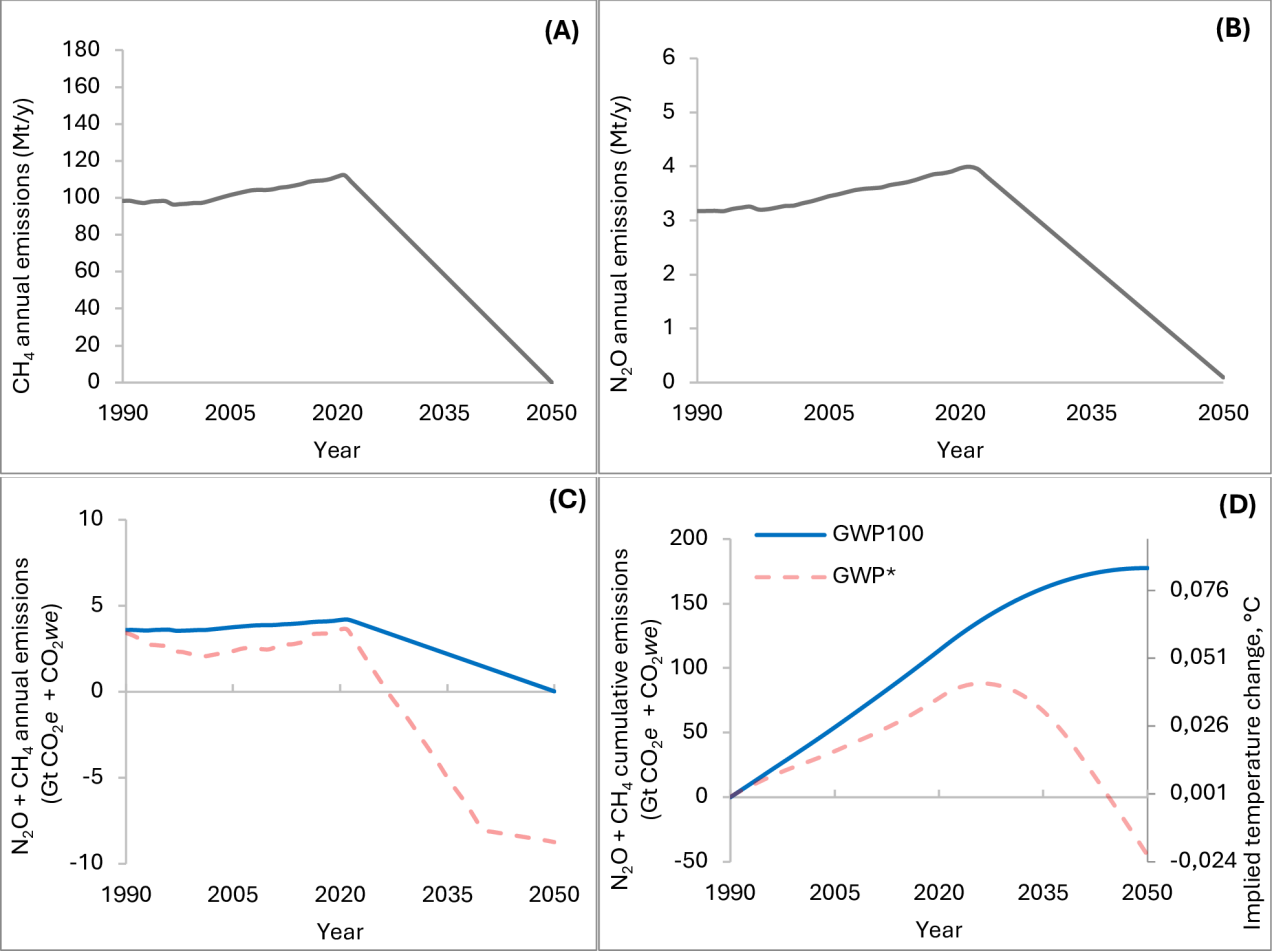
No additional warming scenarios based on GWP_100_. Annual CH_4_ (methane, A) and N_2_O (nitrous oxide, B) emissions rates (Mt/y), corresponding aggregated (CH_4_ + N_2_O) GWP_100_ and GWP* (Gt/y of CO_2_*e* [blue solid line] and CO_2_*we* [light red dashed line]) **(C)**, aggregated cumulative GWP_100_ and related temperature outcomes (Gt of CO_2_*e* and °C [blue solid line]) and aggregated cumulative GWP* and related temperature outcomes (Gt of CO_2_*we* and °C [light red dashed line]) **(D)**, from 1990 to 2050, calculated using the TCRE coefficient (0.45°C/TtCO2e). Abbreviations: GWP* = Global Warming Potential Star; CO_2_*e* = CO_2_-equivalents; CO_2_*we* = CO_2_-warming equivalents; TCRE = Transient Climate Response in Cumulative Emission.

**Fig 4:**
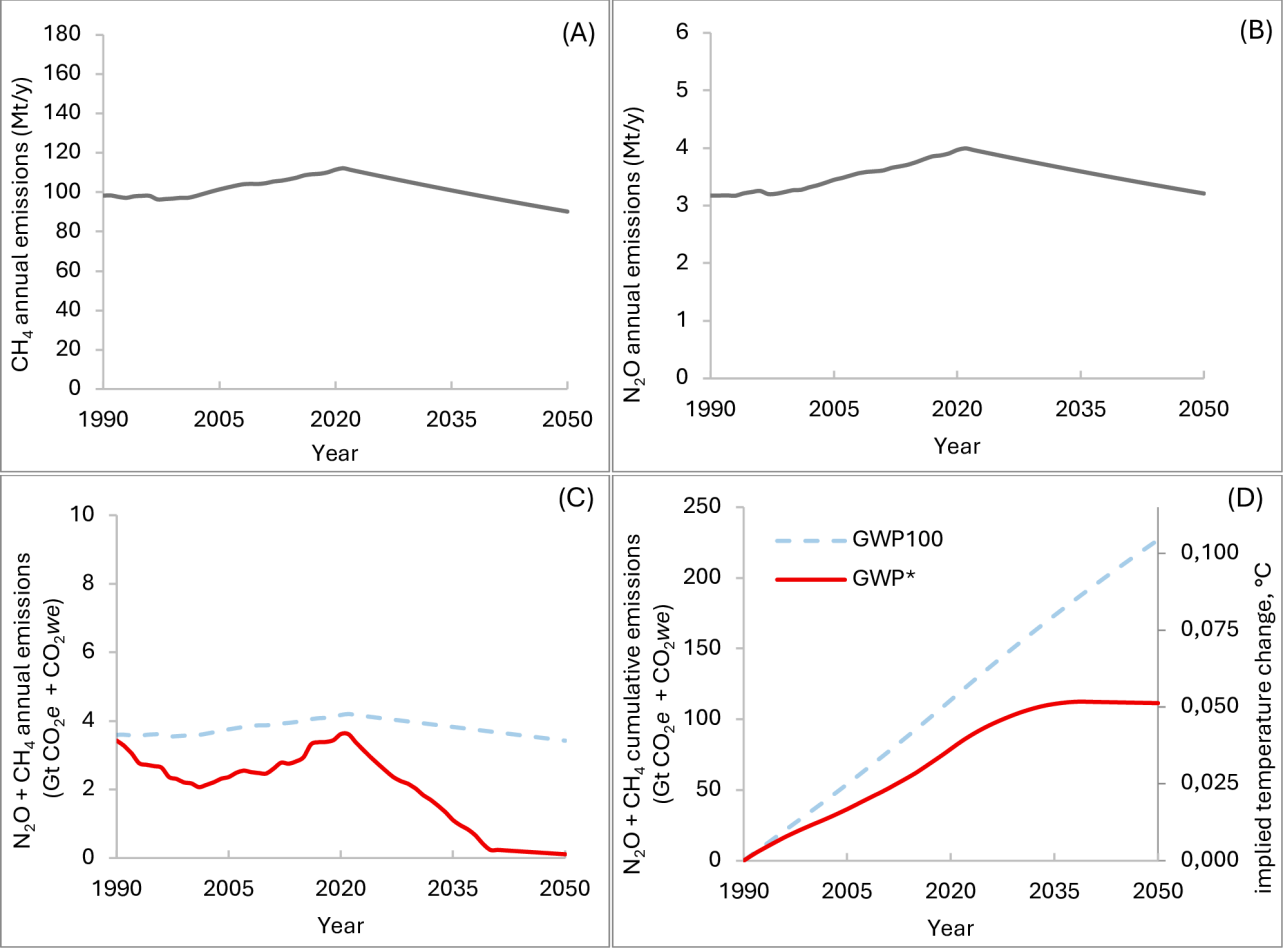
No additional warming scenarios based on GWP*. Annual CH_4_ (methane, A) and N_2_O (nitrous oxide, B) emissions rates (Mt/y), corresponding aggregated (CH_4_ + N_2_O) GWP_100_ and GWP* (Gt/y of CO_2_*e* [light blue dashed line] and CO_2_*we* [red solid line]) **(C)**, aggregated cumulative GWP_100_ and related temperature outcomes (Gt of CO_2_*e* and °C [light blue dashed line]) and aggregated cumulative GWP* and related temperature outcomes (Gt of CO_2_*we* and °C [red solid line]) **(D)**, from 1990 to 2050, calculated using the TCRE coefficient (0.45°C/TtCO2e). Abbreviations: GWP* = Global Warming Potential Star; CO_2_*e* = CO_2_-equivalents; CO_2_*we* = CO_2_-warming equivalents; TCRE = Transient Climate Response in Cumulative Emission.

First, for each considered scenario (increasing, constant, and decreasing), we estimated the CDR required to offset the GHG emissions of livestock exceeding the no additional warming scenarios, by 2050, used to define the “net zero warming goal”. These scenarios were calculated using GWP_100_ (Fig 3) and using GWP* (Fig 4). The “no additional warming scenario” calculated with GWP_100_ (Fig 3) implies a linear reduction of both CH_4_ and N_2_O emissions (Fig 3 A and Fig 3B, respectively), decreasing from 112 to 0 Mt/y and from 4.0 to 0 Mt/y, respectively, from 2021 to 2050. This corresponds to a decrease in terms of aggregated CO_2_*e* from 4.08 to 0 Gt/y (Fig 3C, blue solid line), and to a “stabilization” of implied temperature at 0.08°C by 2050 (Fig 3D, blue solid line). The “no additional warming scenario” calculated with GWP* (Fig 4), is based on a linear reduction of both CH_4_ and N_2_O emissions (Fig 4A and Fig 4B, respectively), from 112 to 94 Mt/y and from 4.0 to 3.0 Mt/y, respectively (from 2021 to 2050). This corresponds to a decrease, in terms of aggregated CO_2_*we,* from 3.37 to 0 Gt/y (Fig 4C, red solid line), and to a “stabilization” of implied temperature at 0.051°C by 2050 (Fig 4D, red solid line).

Figs 3 and 4 report also the aggregated CO_2_*we* and CO_2e_, the cumulative emissions, and temperature outcomes calculated with the alternative metric applied on the same emission trajectories (light red, Fig 3, and light blue, Fig 4, dashed lines, GWP* and GWP100, respectively).

To estimate the required CDR, we tested two metrics, using GWP_100_ or GWP*, according to Brazzola et al. [9].

(1) Using GWP_100_: CDR rates E_CDR_ in (GtCO_2_/y) were fixed equal to CO_2_*e* of livestock emissions of a *i* GHG (CH_4_ or N_2_O) at every point in time (from 2021 to 2050), using GWP_100_ values of 28 and 265 CO_2_*e* for CH_4_ and N_2_O, respectively [19]. The CDR was calculated from the difference in emissions between each of the three considered scenario ${(E}_{scenario,i})$ and the reference scenario: no additional warming scenario ${(E}_{no\;additional\;warming,i})$ that, in this case, corresponds to a net-zero emission scenario; the obtained values were then converted in GWP_100_ values:

$$E_{CDR}=-\Sigma {GWP}_{100,i}\times (E_{scenario,i}-E_{no\;additional\;warming,i})$$

(2) Using GWP*: we set CDR rates E_CDR_ in (GtCO_2_/y) equal to CO_2_*we* of CH_4_ and N_2_O livestock emissions, at every point in time (from 2021 to 2050), using GWP* (refined version of Smith et al. [21]) for CH_4_ and GWP_100_ for N_2_O (values of 28 and 265 CO_2_*e* for CH_4_ and N_2_O, respectively, IPCC, [19]). The CDR was calculated from the difference in emissions between each of the three considered scenario ${(E}_{scenario,i})$ and the reference scenario: the no additional warming ${(E}_{no\;additional\;warming,i})$. It is important to note that under GWP*, no additional warming does not require net-zero emissions. Because GWP* accounts for the ongoing removal of CH₄ from the atmosphere, stable CH₄ emissions can be consistent with constant global temperature, implying that net-zero emissions are not necessary to achieve net-zero warming. Obtained values were then converted in GWP* values:

$$E_{CDR}=-\Sigma {GWP}_{i}^{*}\times (E_{scenario,i}-E_{no\;additional\;warming,i})$$

The overall approach used in this work was similar to that of Brazzola et al. [9], except for the use of the reference scenario that, in our case, is a no additional warming scenario based on GWP_100_ (Fig 3) or GWP* (Fig 4).

## Results

The first results concern the use of two different metrics to describe the temperature impact of global livestock methane and nitrous oxide emissions, under different FAO scenarios by 2050.

**Fig 5:**
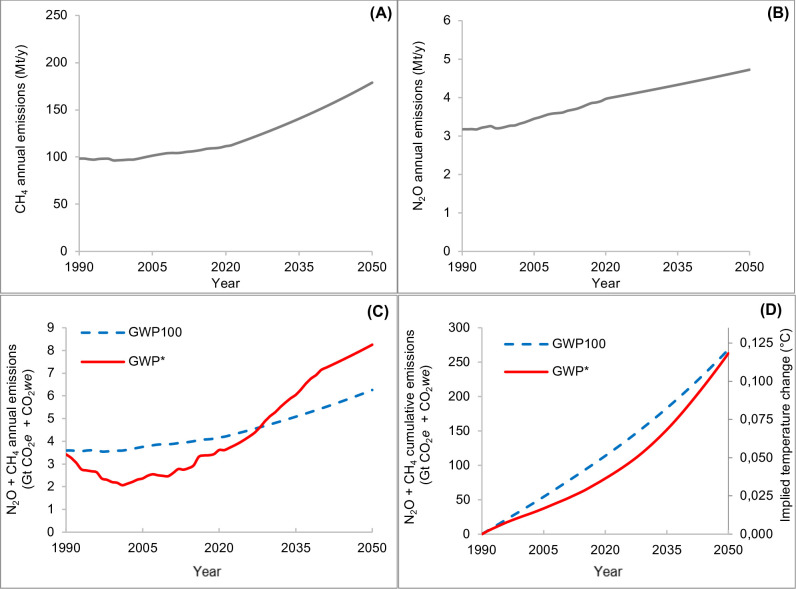
No-mitigation (increasing) scenario. Annual CH_4_ (methane, A) and N_2_O (nitrous oxide, B) emissions rates (Mt/y), corresponding aggregated (CH_4_ + N_2_O) GWP_100_ and GWP* (Gt/y of CO_2_*e* [blue dashed line] and CO_2_*we* [red solid line]) **(C)**, aggregated cumulative GWP_100_ and related temperature outcomes (Gt of CO_2_*e* and °C [blue dashed line]) and aggregated cumulative GWP* and related temperature outcomes (Gt of CO_2_*we* and °C [red solid line]) **(D)**, from 1990 to 2050. Abbreviations: GWP_100_ = 100-year Global Warming Potential; GWP* = Global Warming Potential Star; CO_2_*e* = CO_2_-equivalents; CO_2_*we* = CO_2_-warming equivalents.

Fig 5 presents the “increasing” scenario which is based on the “projected 2050 emissions, no mitigation” of the FAO [13]; in this situation, CH_4_ emissions increase from 112 to 179 Mt/y (Fig 5A), and N_2_O emissions from 4.0 to 4.7 Mt/y, from 2021 to 2050 (Fig 5B).

Fig. 5C shows the aggregated annual emissions from 1990 to 2050, expressed as CO_2_*e* or CO_2_*we*, with the latter being lower than the former in 2028 and then progressively higher. Fig. 5D reports, over the same period, the aggregated cumulative emissions expressed as CO_2_*e* and CO_2_*we* and the related estimated temperature change. In this scenario, GWP_100_ shows increased values of CO_2_*e* annual emission rates of about 50% for the period 2021–2050 (from 4.2 to 6.3 Gt of CO_2_*e*). Following the cumulative CO_2_*e*, the temperature increases reach 0.121°C, a value close to that obtained when considering cumulative CO_2_*we* (0.118°C).

**Fig 6:**
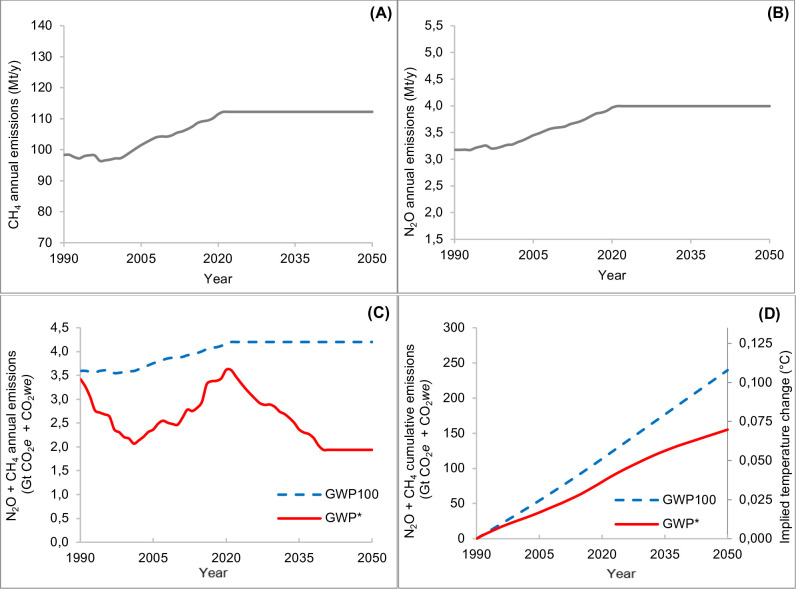
Constant emissions scenario. Annual CH_4_ (methane, A) and N_2_O (nitrous oxide, B) emissions rates (Mt/y), corresponding aggregated (CH_4_ + N_2_O) GWP_100_ and GWP* (Gt/y of CO_2_*e* [blue dashed line] and CO_2_*we* [red solid line]) **(C)**, aggregated cumulative GWP_100_ and related temperature outcomes (Gt of CO_2_*e* and °C [blue dashed line]) and aggregated cumulative GWP* and related temperature outcomes (Gt of CO_2_*we* and °C [red solid line]) **(D)**, from 1990 to 2050. Abbreviations: GWP_100_ = 100-year Global Warming Potential; GWP* = Global Warming Potential Star; CO_2_*e* = CO_2_-equivalents; CO_2_*we* = CO_2_-warming equivalents.

Fig 6 shows an intermediate “constant” scenario, assuming that CH_4_ and N_2_O emissions remain constant until 2050, at the same level as in 2021 (Fig 6A and 6B). Constant emissions result in different pathways between aggregated CO_2_*e* and CO_2_*we* (Fig 6C)*,* the former being flat, mirroring the constant emission pathway, whereas the latter follows a decreasing trend from 2021 to 2040, then remains constant. From 2041 to 2050, GWP_100_ shows values more than double that of GWP* (4.20 CO_2_*e* and 1.94 CO_2_*we*). Looking at the cumulative values (Fig 6D), both the GWP_100_ and GWP* follow an increasing pathway, although to a different extent. The CO_2_*we* increases less than CO_2_*e*, resulting in a lower implied impact on the temperature (in 2050 + 0.070 °C for GWP* and +0.108°C for GWP_100_).

**Fig 7:**
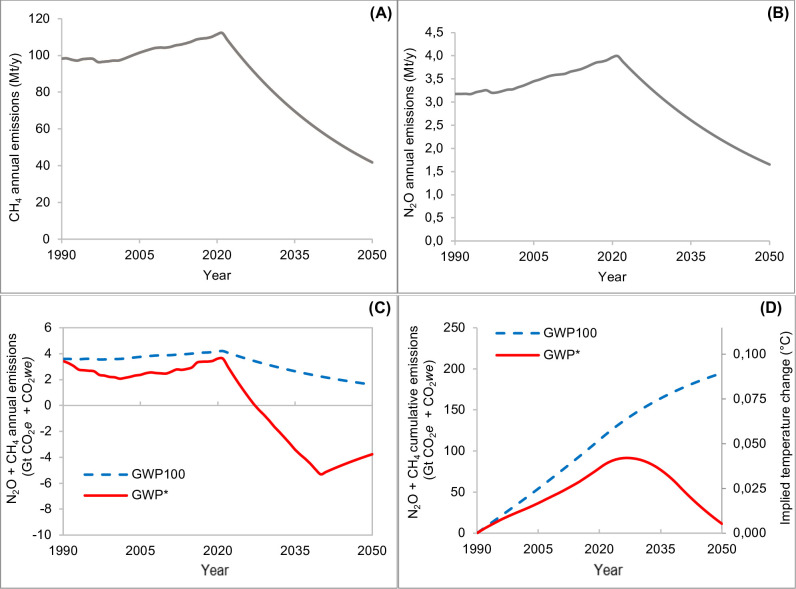
Mitigation (decreasing) scenario. Annual CH_4_ (methane, A) and N_2_O (nitrous oxide, B) emissions rate (Mt/y), corresponding aggregated (CH_4_ + N_2_O) GWP_100_ and GWP (Gt/y of CO_2_*e* [blue dashed line] and CO_2_*we* [red solid line]) **(C)**, aggregated cumulative GWP_100_ and related temperature outcomes (Gt of CO_2_*e* and °C [blue dashed line]) and aggregated cumulative GWP* and related temperature outcomes (Gt of CO_2_*we* and °C [red solid line]) **(D)**, from 1990 to 2050. Abbreviations: GWP_100_ = 100-year Global Warming Potential; GWP* = Global Warming Potential Star; CO_2_*e* = CO_2_-equivalents; CO_2_*we* = CO_2_-warming equivalents.

The “decreasing” scenario (Fig 7) is based on the “Low-emission pathways to 2050” of the FAO [13]. Fig 7 (A and B) reports the CH_4_ and N_2_O, respectively, annual emission rates from 1990 to 2050 (FAO). The GWP_100_ metric shows values of CO_2_*e* emission rates more than halved for the period 2021–2050 (from 4.2 Gt to 1.7 Gt of CO_2_*e*; Fig 7C). Despite this significant drop, the total cumulative emissions still increase over the same timeframe (Fig 7D), reaching slightly less than 200 Gt of CO_2_*e*. Over the same period, these emissions would result in a temperature increase of approximately 0.09°C if they were released as CO₂ (Fig 7D). In contrast, GWP* shows a different pattern; total cumulative emissions (expressed as CO_2_*we*) decrease following the reduction pathways of the two gases (mainly related to the rarefaction of methane, despite continuous accumulation of N_2_O) reaching a value of around 12 Gt of CO_2_*we* by 2050. Consistently, the implied temperature impact, relative to 1990 levels, increases to +0.04 by 2025/26, then decreases, returning close to the initial level (+0.005 °C) by 2050.

The second result relates to the two different metrics used to estimate the carbon dioxide removal needed to offset the excess global livestock methane and nitrous oxide emissions, relative to a no additional warming scenario reached by 2050.

**Fig 8:**
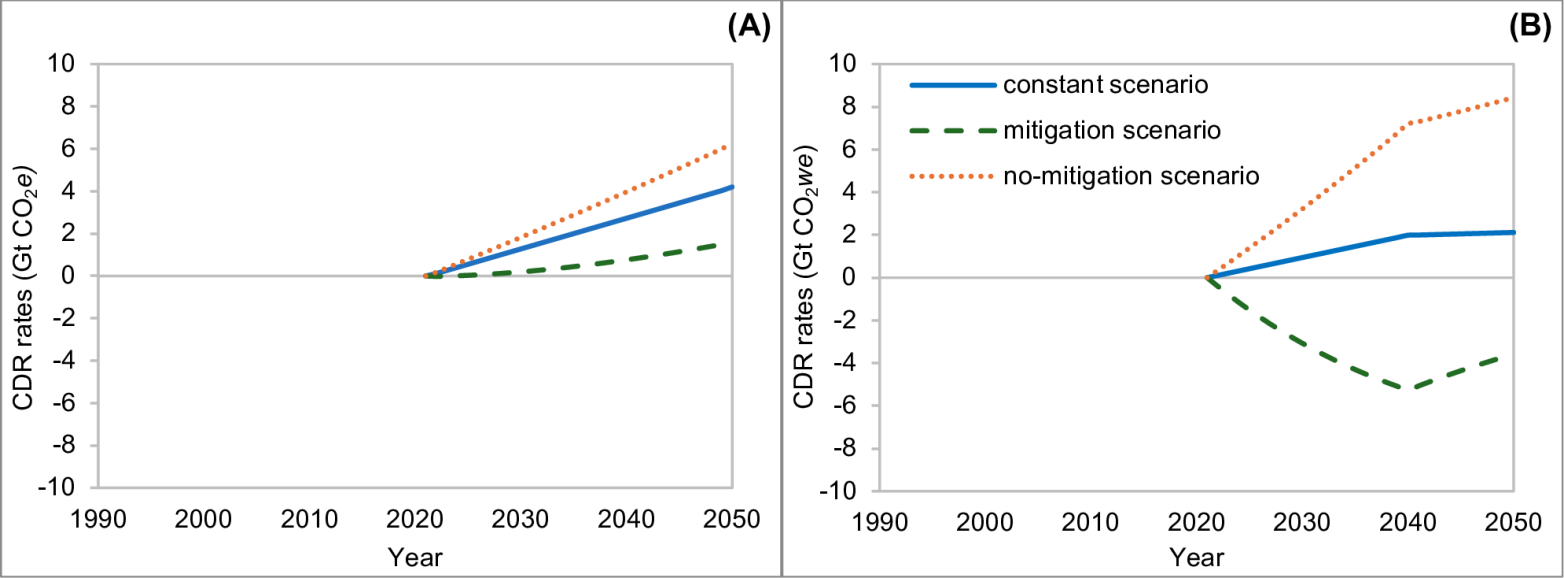
Calculated CDR rates. CDR required to offset global livestock CH_4_ (methane) and N_2_O (nitrous oxide) emissions under the three different scenarios to reach the net zero warming (no additional warming scenario) by 2050, using the GWP_100_ (A) and the GWP* (B) approaches. Constant scenario: green solid lines; mitigation scenario: blue dashed lines; no-mitigation scenario: orange dotted lines. Abbreviations: CDR = Carbon Dioxide Removal; GWP_100_ = 100-year Global Warming Potential; GWP* = Global Warming Potential Star.

Fig 8 reports the calculated CDR rates required to offset global livestock CH_4_ and N_2_O emissions, under the three different scenarios to reach the no additional warming goal by 2050, using the GWP_100_ (A) and GWP* (B). For the GWP_100_ approach, to offset the exceeding emissions in the constant scenario we will need to increase the CDR to reach a value of 4.2 GtCO_2_e by 2050. As far as the increasing (no mitigation) scenario is concerned, the increase of CDR will reach the value of 6.26 GtCO_2_e by 2050. The mitigation scenario shows still greater annual CDR values compared to no additional warming scenario, reaching the maximum value of 1.61 GtCO_2_e by 2050.

Under the GWP* offsetting approach, to offset the exceeding emissions in the constant scenario we would require an increase in annual carbon sequestration to 1.83 GtCO_2_*we* by 2050. Looking at the increasing (no mitigation) scenario, the rise in carbon sequestration will reach the value of 8.14 GtCO_2_*we* in 2050. The mitigation scenario shows negative values of CRD, reaching −3.9 GtCO_2_*we* in the 2050.

## Discussion

This work aims to highlight how the increasing demand for animal-derived foods can be met while coping with the temperature goal achievement. This is possible by implementing all the feasible strategies for reducing GHG emissions and enhancing removal by sinks. However, this statement is not so clear without the application of appropriate metrics.

The examples provided in this work show how the new metric GWP* gives different results compared to the GWP_100_. This implies the adoption of different climate mitigation strategies to achieve the temperature goal. According to previous works highlighting the inappropriateness of standard GWP_100_ to study the climate impact of these GHGs under historical and future emissions scenarios [4,25,26], the GWP* should be used to better evaluate the impact of SLCPs.

The main problem of using GWP_100_ to describe the climate impact of SLCPs is related to the conversion of emissions to temperature outcomes. As can be deduced from the examples, the ability of GWP_100_ to capture the more realistic temperature change depends on the emission scenario.

It should be emphasized that specific climate models exist to estimate the temperature outcomes of GHG emissions. However, in this study we use a simpler approach for this aim, based on the near linearity of cumulative CO_2_ emissions with temperature change (TCRE). TCRE is a simplified method of temperature outcome estimation, which provides a good approximation for the warming impact of LLCFs but fails to reflect the warming impact of SLCFs [3] when their emissions are reported as CO_2_*e*. The present work aims to show how the application of different metrics to livestock emission scenario may have huge influence in the interpretation of the temperature outcomes and, consequently, can have a major impact on future policies related to the sector.

Looking at the results, in the increasing scenario the impact of GHG emissions on the implied temperature change is similar between the two metrics (0.121°C and 0.118°C for GWP_100_ and GWP*, respectively), whereas it diverges for the constant emission scenario (0.108°C for GWP_100_ and 0.070 °C for GWP*) and it assumes a huge difference in the decreasing scenario, ranging from 0.04 °C with GWP100 to 0.005 °C with GWP*.

**Fig 9:**
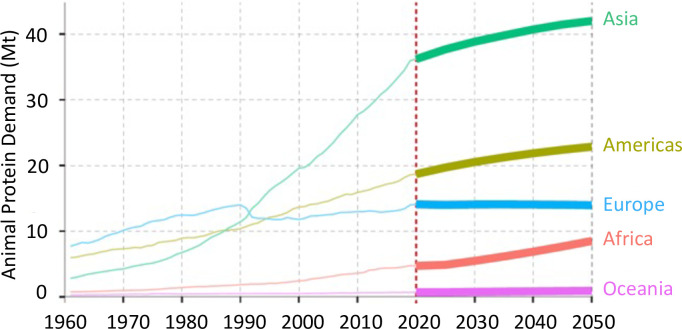
Projected animal protein demand in different regions by 2050.

Adapted from Fig 11 of the FAO report [13].

The increasing scenario assumes that the emissions of CH_4_ and N_2_O will rise following the needs of the growing population, without the application of any mitigation strategy. This scenario was proposed by the FAO [13] considering the business-as-usual (BAU) scenario [27] which estimates rising demand for animal protein, expected to be globally + 21% by 2050. Demand for animal protein will be different depending on the continent considered (Fig 9): lowest relative change is expected in Europe (+1%) and the highest in Africa (+102%); Asia will account for +18% of relative change, but it will experience the highest absolute increase (42.57 Mt of terrestrial ASF proteins by 2050).

The results of the increasing emission scenario (Fig 5) reveal that GWP_100_ underestimates the warming impact of rising CH_4_ emissions. This was expected as an annual increase in CH_4_ greater than 1% results in higher CO_2_*we* values than CO_2_*e* values [6]. Similar outcomes were previously observed by other authors [9,28,29]. In this study, due to the rate of emissions increase of the BAU scenario, the temperature outcomes were similar across metrics by 2050 (0.118 and 0.121 for GWP* and GWP100, respectively). Del Prado et al. [4] reported greater temperature impact of GWP* compared to GWP_100_, when the Brazilian cattle CH_4_ emissions trend was considered.

The constant and decreasing scenarios reveal how the GWP_100_ fails to convert the emission pattern into a realistic temperature outcome. In the first case, GWP_100_ overestimates the effect on temperature of about 0.04°C by 2050, compared to GWP* estimates. In the second case, despite a continuous reduction of GHG, the implied temperatures still increase to about 0.09 °C by 2050; on the other hand, the same emissions pattern, when assessed using GWP*, results in a stabilization effect, starting from 2025 (0.042°C), and a cooling effect, from 2030, reaching the value of 0.005 °C by 2050. Regarding the decreasing scenario, it should be highlighted that it describes an optimistic situation and has different limitations. It assumes that the impact of mitigation interventions is cumulative and with no overlaps. Unfortunately, it is well known that some of the proposed interventions involves intricate interactions and interdependencies. As an example, the livestock productivity can be enhanced by different interventions, as feeding strategies, selective breeding, or improvement of health and welfare of animals [30]. On the other hand, it should be highlighted that all the strategies reported in the “low emission scenario” are based on scientific literature, and the impact on the emissions decrease is highly confident, considering that the effect of some of them has been substantial over the last decades and well documented [13]. According to the FAO & GDO [31] between 2005 and 2015 the overall milk production has increased by 30% while, at the same time, the global dairy herd increased 11%. The growth in farm and animal production efficiency has allowed to limit the increase of global dairy sector GHG emissions from an expected 38% to a 18%, in the same time frame. This trend is the result of different efficient improvement strategies, and it is mainly driven by the developed countries as those of north America and west Europe. In the EU, in 2 decades from the 2002–2022, the dairy cow milk production increased by 15.7% while the number of dairy cows decreased by 16.9% [32]. In terms of emission intensity (calculated from the total CH_4_ and N_2_O emissions of the EU dairy cattle sector), it means a reduction from 0.82 to 0.58 kg CO_2_*e*/kg of milk. Furthermore, it should be hypothesized that the incessant research, aimed at reducing the livestock GHG emissions, will bring novel or improved strategies by 2050, allowing to reach or overcome the optimistic “low emission pathways” reported in the FAO report.

The comparison between results from GWP_100_ and GWP* has significant implications for net-zero strategies, as discussed below. Our analysis compares the estimated CO₂ removal requirements under both metrics, demonstrating how the metric choice affects net-zero strategies and the estimated temperature outcomes. It should be highlighted that we do not claim to validate a climate model but rather apply established methodologies for estimating temperature responses based on cumulative emissions. As recognized in IPCC AR6 Chapter 7 (WGI) [5], “GWP* better represents the climate impact of methane over time” compared to GWP_100_, particularly when emissions are changing [5,33,34]. Moreover, it has previously demonstrated that when the separate gases are inputted into a model, the temperature outcomes align with those indicated by GWP* [4,33]. Our results highlight the implications of metric selection for climate mitigation strategies, emphasizing the need for policy frameworks that consider the most appropriate methods for short-lived climate pollutants.

To reach the temperature goal (no additional warming), deliberate carbon dioxide removal (CDR) from the atmosphere is essential to offset residual GHG emissions [5]. In this context, the choice of emissions metric affects the quantification of temperature outcomes of GHG emissions, for achieving and sustaining a condition of no additional warming [5]. Indeed, a key difference between the two metrics can also result from analyzing the two no additional warming scenarios (Fig 3 and 4), used as reference scenarios, to calculate the CDR. The achievement of no additional warming would imply different targets for different GHG [4]: for the CO_2_-induced warming (or if we use a GWP_100_ metric to build a no additional warming scenario) all the emissions should be decreased to zero (net-zero emissions, Fig. 3 A, B for CH_4_ and N_2_O annual emissions, and C blue solid line for the aggregate CO_2_*e*); however, this is not realistic for the SLCP; indeed, for CH_4_, even ongoing emissions can be compatible with no additional warming, considering that past emissions are constantly removed from the atmosphere. Consequently, the achievement of temperature goal does not necessarily coincide with net-zero CH_4_ emissions, but rather CH_4_ emission pattern that does not generate additional warming, as can be observed for the scenario calculated with GWP* (Fig. 4). As far as the net zero warming goal is concerned, it should be noticed that there is no officially established “net zero warming” goal; rather, this no additional warming scenario was proposed as reference scenario in this work, with the only aim to better explore the different implications of using the two metrics when calculating the amount of emissions to be offset in order to achieve the climate goal. This reference scenario is in some way similar to that used by Brazzola et al. [9] “SSP1-2.6”. In that scenario, the reduction of methane emissions from the agricultural sector (by 56% by 2100 from their observed values in 2015) would lead to keeping the temperature increase within the range of 1.5–2 °C, by 2100.

Regarding the approaches used for the quantification of CDR, our results highlight the unsuitability of the widely used GWP_100_, in accordance with previous observations by Brazzola et al. [9].

It should be noted that all the results of this investigation are related to the aggregated emissions of the two considered GHGs, CH_4_ and N_2_O, but are driven by the CH_4_ emission pattern. In this context, the IPCC metric underestimates the amount of CDR needed to offset increasing emissions of SLCP, and overestimates CDR under constant and decreasing emissions scenarios. The differences between the two approaches are not negligible. Under the increasing scenario, GWP_100_ underestimates the CDR compared to GWP* (6.26 vs 8.14 GtCO_2_/y at the end of the considered period). On the other hand, GWP_100_ overestimates the CDR under the constant scenario (4.2 vs 1.83 GtCO_2_/y, by 2050) and provides opposite indications regarding the mitigation scenario; in this last case, the value obtained using the GWP_100_ approach is 1.6 GtCO_2_/y by 2050, whereas that obtained with the GWP* is −3.87 GtCO_2_/y. The mitigation scenario shows negative values of CDR, indicating a more favorable scenario also compared to the no additional warming one. In this case the global livestock systems will have a large amount of carbon credits (potentially reaching 1.76 GtCO_2_/y).

These results should place even greater emphasis on the importance of improving and promoting all the possible mitigation strategies. The high rate of CDR resulting from the increasing (no-mitigation) scenario would entail a very big effort to offset incremental emissions. Moreover, many of the CDR techniques are still not well established from scientific and practicability perspectives, and the cost and social implications of such strategies are also not negligible [35,36]. It should be stressed that several of the possible interventions are typically capital-intensive, geographically constrained, and not achievable at local scales (e.g., direct air carbon dioxide capture and storage, bioenergy with carbon capture and storage, and ocean fertilization; [35]); for this reason, polluters (entrepreneurs or countries) should pay a tax to cover the costs for CDR related to their activities. This poses an important ethical issue, considering that the main increase in livestock emissions by 2050 is expected to arise from developing countries (FAO report), which suffer from an economic and technological gap compared to the developed countries. In this context, an international government of CDR and a more equitable allocation of related costs are desirable but lacking. Despite CDR being under continuous studies and scientific debate and it is recognized being able to play an important role in achieving the 1.5°C goal, it is not yet firmly on national or global policy agendas [37,38]. In the livestock sector, the discussion of CDR as mitigation option is almost limited to the local scale interventions, as soil carbon sequestration [39,40]. In this context, direct mitigation interventions (as reported in the FAO report) could avoid high rates of CDR (even negative values estimated in the mitigation scenario, Fig. 8) and can represent the most suitable way to reduce livestock climate impact, from a practical and economical point of view. Indeed, most of the emissions reduction strategies can be implemented using existing knowledge and technologies and, often, can provide increase in productivity and profitability [41]. The effectiveness of these measures has been widely documented, and their marginal abatement costs are often low or even negative, particularly when implemented as part of integrated mitigation packages [42]. Precision livestock farming and digital technologies can play an important role in supporting mitigation strategies and value chain improvements in the sector [43,44]. However, special effort should be made to promote and develop these technologies also in developing countries or regions that are still facing difficulties from an infrastructure point of view [45]. As highlighted by Vuvor et al. [46], the adoption of digital tools, especially mobile-based platforms and SMS services, is already improving access to market information, buyer networks, quality control, and supply chain transparency in several African countries. These tools not only help producers increase efficiency and income but also enable more precise monitoring and coordination of emissions reduction efforts. Although challenges remain, such as limited connectivity, high device costs, and disparities in digital literacy, the ongoing digital transition in many developing countries represents a promising frontier for sustainable intensification and for the implementation of verifiable in-setting strategies, whereby climate benefits are generated and retained within the same production systems.

## Conclusions

The use of the GWP* metric compared to the IPCC’s GWP metric, applied to the FAO scenarios, for assessing the carbon offsets needed to cancel out livestock emissions by 2050, yields different results. In the case where the increase in emissions follows that of ASF demand, the former underestimates the required offset relative to the latter, while in the case of emissions mitigation it goes so far as to assess carbon credits instead of debts. In terms of implied temperatures, calculated on the basis of 1990, the two metrics do not deviate in the worst-case scenario of business-as-usual, but provide opposite results in the best-case scenario of emissions mitigation. These results show the need to use the GWP* metric to better meet the temperature goal by acting in particular on the SLCP represented by methane, the main GHG emitted by livestock supply chains worldwide. Finally, the results of this work highlight that for livestock is not mandatory to reach net-zero emissions to achieve temperature goals; rather, the livestock sector, by adopting possible mitigation strategies, can play a crucial role in limiting climate change, while ensuring an increased global supply of animal products by 2050.

## Supporting information

S1 Data Supporting data Correddu et al. (XLSX)
